# KIDMAP, a web based system for gathering patients' feedback on their doctors

**DOI:** 10.1186/1471-2288-9-38

**Published:** 2009-06-17

**Authors:** Tsair-Wei Chien, Weng-Chung Wang, Sho-Be Lin, Ching-Yih Lin, How-Ran Guo, Shih-Bin Su

**Affiliations:** 1 Department of Management, Chi-Mei Medical Center, Tainan, Taiwan; 2 Department of Hospital and Health Care Administration, Chia-Nan University of Pharmacy and Science, Tainan, Taiwan; 3 Department of Educational Psychology, Counseling and Learning Needs, Hong Kong Institute of Education, Hong Kong, PR China; 4 Department of Internal Medicine, Chi-Mei Medical Center, Tainan, Taiwan; 5 Department of Environmental and Occupational Health, National Cheng Kung University, Tainan, Taiwan; 6 Department of Biotechnology, Southern Taiwan University, Tainan, Taiwan; 7 Tainan Science Industrial Park Clinic, Chi-Mei Medical Center, Tainan, Taiwan

## Abstract

**Background:**

The gathering of feedback on doctors from patients after consultations is an important part of patient involvement and participation. This study first assesses the 23-item Patient Feedback Questionnaire (PFQ) designed by the Picker Institute, Europe, to determine whether these items form a single latent trait. Then, an Internet module with visual representation is developed to gather patient views about their doctors; this program then distributes the individualized results by email.

**Methods:**

A total of 450 patients were randomly recruited from a 1300-bed-size medical center in Taiwan. The Rasch rating scale model was used to examine the data-fit. Differential item functioning (DIF) analysis was conducted to verify construct equivalence across the groups. An Internet module with visual representation was developed to provide doctors with the patient's online feedback.

**Results:**

Twenty-one of the 23 items met the model's expectation, namely that they constitute a single construct. The test reliability was 0.94. DIF was found between ages and different kinds of disease, but not between genders and education levels. The visual approach of the KIDMAP module on the WWW seemed to be an effective approach to the assessment of patient feedback in a clinical setting.

**Conclusion:**

The revised 21-item PFQ measures a single construct. Our work supports the hypothesis that the revised PFQ online version is both valid and reliable, and that the KIDMAP module is good at its designated task. Further research is needed to confirm data congruence for patients with chronic diseases.

## Background

The consumerist approach to health care [1] requires doctors to be more accountable to their patients [2-4]. Patient-centered care is widely recognized and has become a key aim of hospitals and healthcare systems in recent years. Accordingly, healthcare service assessment at a general level, namely within a hospital, or within a particular kind of healthcare service, is needed and the patient feedback survey is an important component of this quality monitoring [5]. However, mechanisms for assessing patient views on performance and practice at the physician level are not as widely established as the systems for gathering feedback from patients at the organization level [6]. The purpose of this study was (a) to establish a valid and reliable instrument for the measurement of physicians' performance, and (2) to develop an effective way to quickly gather feedback on doctors from patients after a consultation.

### Increasing importance of patient evaluation of physician performance

Physicians play a key role in the overall quality of patient care. Feedback after consultations helps identify strengths and weaknesses at the level of the doctor's practice, and directs them to areas where improvement is required [7]. Many hospital initiatives use questionnaires to assess satisfaction with doctors' performance as part of routine management [8]. These questionnaires draw attention to issues such as the doctor's communication skills in order to improve the quality of medical practice effectively [9,10]. The assessment of individual doctor performance has thus gained increasing prominence worldwide [11].

### Web- KIDMAP to gather feedback efficiently from patients

Two new modes of administration, using automated technology to complete questionnaires over telephone through interactive voice response (IVR) and using the Internet-like visualization to complete questionnaires on-line, make surveys more easily accessible to those who do not read or write [12]. Rodriguez et al. [13] and Leece et al. [14] observed that a Web survey produces an approximately equal response rate to a mail survey. Ritter et al. [12] also observed that not only is Web survey participation at least as good as mail survey participation, but Internet questionnaires require less follow-up to achieve a slightly, but non-significantly higher completion rate than mailed questionnaires [15].

Web surveys have the advantage that respondents can remain anonymous [16]. In addition, patients benefit from the Internet as it is being used [17]. They can acquire additional information, advice and social support from the Internet. Furthermore, Internet information can be directly stored in a database and is immediately accessible for analysis. Undoubtedly, web-based feedback will begin to prevail in the era of the Internet [18]. A simpler, faster and cheaper way of gathering feedback from patients is thus encouraged by caregivers.

In the past, however, most patient questionnaires are generally of a paper-and-pencil format. Questionnaires are usually distributed using either a consecutive sample or a random sample and the respondents usually return questionnaires either in person or by mail. These traditional methods do not allow for simultaneous data processing and analysis.

Furthermore, most data analysis of patient questionnaires is based on the classic test theory (CTT). In recent years, the CTT has been gradually replaced by the item response theory (IRT) [19,20]. This study shows how to apply IRT to fit questionnaire data from patients and develops a web version of KIDMAP [21,22] to help doctors easily and quickly summarize individual patient satisfaction levels and identity aberrant responses.

### Reasons for the use of the IRT 1-parameter Rasch model for KIDMAP

IRT was developed to describe the relationship between a respondent's latent trait (namely performance by the service provider or satisfaction with the service provider in this study) and the response to a particular item. A variety of models have been proposed, including the 1-, 2- and 3-parameter logistic models, of which the 1-parameter model is also referred to as the Rasch model. All three models assume a single underlying continuous unbounded variable designated as ability for the respondents, but which varies in the characteristics they ascribe to items. All three models have an item difficulty parameter, which is the point of inflection on the latent trait scale.

The Rasch model has some advantages over the 2- and 3-parameter models [19-22]. The Rasch model lends itself to a total summed score as a sufficient statistic for ability estimation, and the summed score of respondents to an item as a sufficient statistic for difficulty estimation. Thus, the model fits nicely with total summed scoring. In addition, respondents with the same raw score will always have the same estimated latent trait level, which is not the case with the 2- and 3-parameter models. Accordingly, the Rasch model was applied to fit the dataset collected in this study, and used to develop the Web-KIDMAP [23,24].

### The Patient Feedback Questionnaire

The Picker Institute Europe [25] developed a 23-item questionnaire to survey "what do you think of your doctor." The questionnaire has been reviewed, refined and tested for validity and reliability using ten selected instruments, but the methodology and results of testing have not yet been published. With permission, the 23-item Patient Feedback Questionnaire (PFQ) was analyzed to show whether they measure a single construct and fit the Rasch model's expectation. After checking model-data fit, we developed the Web-KIDMDAP to summarize individual patient results and implemented it using email.

## Methods

### Participants and Procedure

The study sample was recruited from outpatient visitors to a 1300-bed medical center in Taiwan. During each interval period in the morning, afternoon, and at night from Monday through Friday in the last week of November 2007, 30 patients who had just finished a consultation with a doctor were selected randomly. A total of 450 respondents either self-completed the questionnaire or had a face-to-face interview if they were unable to personally complete the questionnaire; no proxies were allowed. This study was approved and monitored by the Research and Ethical Review Board of the Chi-Mei Medical Center.

### Instrument and measures

The 23-item PFQ [26] consists of five domains: interpersonal skills (5 items), communication of information (4 items), patient engagement and enablement (7 items), overall satisfaction (3 items), and technical competence (4 items) [see Additional file 1]. Each item is assessed using a 5-point Likert scale (ranging from strongly disagree to strongly agree). The WINSTEPS computer program [27] was used to fit the Rasch rating scale model [28], in which all items shared the same rating scale structure [29].

### Data Analysis

The analysis was composed of two parts. The model-data fit was assessed by item fit statistics and analysis of differential item functioning [30,31] across the subgroups of patients. An illustration of the Web-KIDMAP showing visual representations of the respondent's views about the doctor performance is also provided.

### Model-data fit

There are two kinds of item fit statistics, un-weighted Outfit and weighted Infit mean square error (MNSQ), which can be used to examine whether items meet the Rasch model's requirement. The Outfit MNSQ directly squares and averages standardized residuals; while the Infit MNSQ averages standardized residuals with weights [19,21]. The MNSQ statistics are Chi-squared statistics divided by their degrees of freedom. The Outfit and Infit MNSQ statistics have an expected value of unity when the data meet the model's expectation [29]. Two major assumptions must hold to yield interval measures. For the assumption of unidimensionality, all items must measure the same latent trait, for example the doctor's performance; a value of MNSQ greater than 1.4 indicates too much noise. For the assumption of conditional (local) independence, item responses must be mutually independent and conditional on the respondent's latent trait. A value of MNSQ less than 0.6 suggests too much redundancy.

For rating scales a range of 0.6 to 1.4 is often recommended as the critical range for MNSQ statistics [21,32]. Items with an Outfit or Infit MNSQ beyond this range are regarded as having a poor fit. It has been argued that the Rasch model is superior to factor analysis in terms of confirming factor structure [33]. When poor-fitting items are identified and removed from the test, unidimensionality is guaranteed and interval measures can be produced.

### Assessment of differential item functioning (DIF)

In order to make a comparison across different groups of respondents, the test construct must remain invariant across groups. DIF analysis is a way of verifying construct equivalence over groups [31]. If construct equivalence does not hold over groups, meaning that different groups respond to individual questions differently after holding their latent trait levels constant, then the estimated measures should not be compared directly over the groups. All items ought to be DIF-free or at least DIF-trivial in order to obtain comparable measures over the groups [31].

An item is deemed to display DIF if the response probabilities for that item cannot be fully explained by the latent trait. DIF analysis identifies items that appear to be unexpectedly too difficult or too easy for a particular group of respondents. Four demographic characteristics were tested for DIF in this study, namely gender (two groups), education (classified into three groups: elementary, secondary, and higher education), age (classified into five groups: 20–29, 30–39, 40–49, 50–59, and over 60), and the type of disease prompting consultation (classified into five categories: internal medicine, surgical medicine, obstetrics & gynecology, pediatric medicine, and others). A difference larger than 0.5 logits (equal to an odds ratio of 1.65) in the difficulty estimates between any of the groups was treated as a substantial DIF [31]. Once found, DIF items were removed from further analysis. The analyses stopped when all the Outfit and Infit MNSQ statistics were located within the (0.6, 1.4) critical range and no further DIF items could be identified.

### Difference between DIF and item misfit

It is important to make a distinction between DIF and item misfit. A misfit item may be due to many causes, such as model misspecification, local dependence or multidimensionality. A DIF item is restricted to what functions are different for the distinct groups of respondents and therefore does not have the same item parameter estimations as the groups.

If an item is found to have a poor fit as a whole or within any group of respondents, it should be removed from the data set. This process ensures that item parameter calibrations obtained from each group are meaningful and comparable. Otherwise, it would be inappropriate to compare the latent trait levels within and between groups.

### KIDMAP development

KIDMAP, developed within the context of Rasch measurement [22], is a method of displaying academic performance. Four quadrants are used: harder items not achieved (i.e., under expectation when the responded score is less than the expected value on the respective items), harder items achieved, easier items not achieved, and easier items achieved. Additionally, respondent errors that require more attention are plotted on the bottom right quadrant. A complete KIDMAP highlights a patient's satisfaction level and pinpoints the strengths and weaknesses of the evaluated doctor performance [34,35].

## Results

### Participants

There were 450 eligible participants of whom five received a perfect or zero total score. They were representative of a national large-size hospital outpatient population in terms of gender, education, age and disease, as shown in Table 1.

**Table 1:** Demographic background of the 445 patients

Characteristics	Number	%	Characteristics	Number	%
***Gender***			***Education***		
female	239	54%	elementary	303	68%
male	189	42%	secondary	85	19%
none or missing	17	4%	higher	46	10%
			none or missing	11	3%
***Age***			***Disease***		
20–29	17	4%	internal medicine	109	24%
30–39	28	6%	surgical medicine	97	22%
40–49	121	27%	O & G*	41	9%
50–59	243	55%	pediatric medicine	25	6%
over 60	14	3%	others	129	29%
none or missing	22	5%	none or missing	44	10%

* O & G: obstetrics and gynecology

### Model-data fit

All but items 22 and 23 had a fit MNSQ in the range between 0.6 and 1.4. These two items were removed. Table 2 shows that the assumption of unidimensionality held for these 21 polytomous items when assessed across the 445 patients. The two items that were removed, which were related to offering chronic and preventive services, were inappropriate to acute patients examined in the study. Difficulty values for the remaining 21 items differed slightly from one another (*M *= 0, *SD *= 0.30). Furthermore, the ordered natures of the category boundary threshold parameters were estimated as -3.06, -2.36, 0.44, and 4.97 under the rating scale model, indicating that no item exhibited disordering of the step difficulty and the 5-point rating scale was appropriate [36,37].

**Table 2:** Difficulty, SE, DIF and fit statistics for the 21-item Patient Feedback Questionnaire

	Difficulty	SE	Classified groups for DIF				MNSQ	
	Logits	Logits	*Gender*	*Education*	*Age*	*Disease*	*Outfit*	*Infit*
Interpersonal skills								
2	0.27	0.10	0.05	0.08	0.36	0.63*	1.05	1.05
4	0.17	0.11	0.00	0.04	0.50	0.35	0.82	0.80
3	-0.01	0.10	0.06	0.01	1.46*	0.38	1.11	1.09
1	-0.11	0.10	0.06	0.08	0.95*	0.34	0.98	1.01
5	-0.52	0.10	0.04	0.08	0.70*	0.34	0.85	0.85
Communication of information								
9	0.51	0.10	0.10	0.08	0.73*	0.14	0.81	0.84
8	0.22	0.10	0.08	0.13	1.09*	0.44	0.90	0.86
6	0.01	0.10	0.13	0.05	0.60*	0.15	0.95	0.90
7	0.00	0.10	0.08	0.06	0.33	0.36	0.88	0.84
Patient engagement and enablement								
9	0.52	0.10	0.06	0.03	0.76*	0.50	1.24	1.24
6	0.3	0.10	0.06	0.09	0.74*	0.75*	0.83	0.80
7	0.1	0.10	0.04	0.04	0.57*	0.34	1.05	1.10
15	0.03	0.10	0.04	0.17	1.25*	0.65*	1.18	1.11
16	-0.13	0.10	0.24	0.09	0.54*	0.31	0.76	0.69
14	-0.21	0.10	0.23	0.19	1.07*	0.77*	1.30	1.22
8	-0.3	0.10	0.00	0.12	0.36	0.19	1.00	0.89
Overall satisfaction								
19	-0.06	0.10	0.11	0.10	0.47	0.24	1.22	1.10
17	-0.26	0.10	0.14	0.02	0.80*	0.33	1.04	1.10
18	-0.69	0.11	0.09	0.05	0.37	0.59*	0.83	0.76
Technical competence								
21	0.27	0.10	0.08	0.08	0.84*	0.59*	1.14	1.12
20	-0.13	0.10	0.03	0.05	0.21	0.85*	1.02	0.95
MEAN	0.00	0.10	0.08	0.08	0.70	0.44	1.00	0.97
S.D.	0.30	0.00	0.06	0.05	0.33	0.21	0.16	0.15

Note: *Substantial DIF (a difference in item difficulties larger than 0.5 logits between groups); *Gender*: 1 = Male, 2 = Female; *Education*: 1 = Elementary, 2 = Secondary, 3 = Higher education; *Age*: 1 = 20–29, 2 = 30–39, 3 = 40–49, 4 = 50–59, 5 = Over 60; *Disease*: 1 = Internal medicine, 2 = Surgical medicine, 3 = Obstetrics & Gynecology, 4 = Pediatric medicine, 5 = Others.

The person measures (ranging from -7.35 to 9.24) had a mean of 3.06 logits and a *SD *of 2.63, indicating that the items were easily satisfied for respondents with an averaged odds ratio of 21.33 (= *e*^3.06^) compared to the average item difficulty with zero logit and that the items were able to group into five strata [19]. The person separation reliability (similar to Cronbach's α) was 0.94, indicating that these items yielded very precise estimates for the patients.

### DIF assessment

DIF analysis was conducted to assess the model-data fit for item-difficulty hierarchy that was invariant across groups. Table 2 lists the maximum differences in the estimates of item difficulty across groups. We took a difference greater than 0.5 logits as a sign of substantial DIF [31]. None of the 21 items for the gender and education groups displayed DIF, but several items displayed DIF for the age and disease groups. These results mean that people from different groups in terms of age or disease with the same latent trait level (ability/satisfaction) have a different probability of giving a certain response on some items. For example, item 6 (giving clear understandable explanations about diagnosis and treatment) had DIF across the age groups. Hence, it is not appropriate using the summed scores or the estimated satisfaction levels to compare age groups against each other.

### KIDMAP demonstration

The KIDMAP output is very intelligible to the patient, his/her doctor or the doctor's staff. It indicates whether the patient's response is reasonable as well as which areas the doctor need to improve and does this by referring to the item scatter on the map. The probability of success on an item is shown on the farthest left-hand side of the map. In the center column "XXX" locates the satisfaction estimate of the patient. Percentile ranks, frequencies and the distribution of the norm-reference from the 445 patients are shown on the right-hand side, but some are omitted for space-saving reasons. Item difficulties and person measures are depicted on an interval continuum scale on the right-hand side. A fit MNSQ larger than 2.0 indicates that the segments of the data may not support useful measurement and that there is more unexplained noise than explained noise. In other words, there is more misinformation than information in the observation [36].

**Figure 1:**
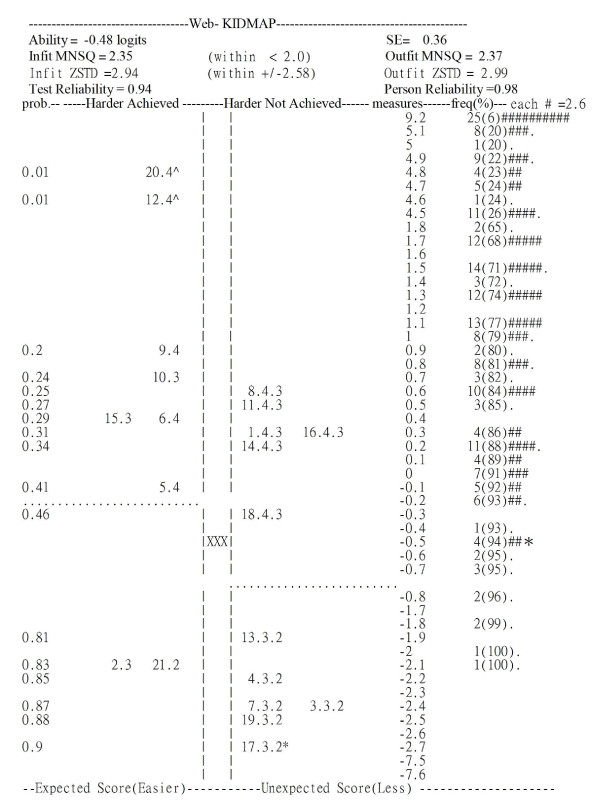
**KIDMAP for patient 1 showing an inadequate fit to the model**. Note: *p < .05; ^ *p *< .01.

**Figure 2:**
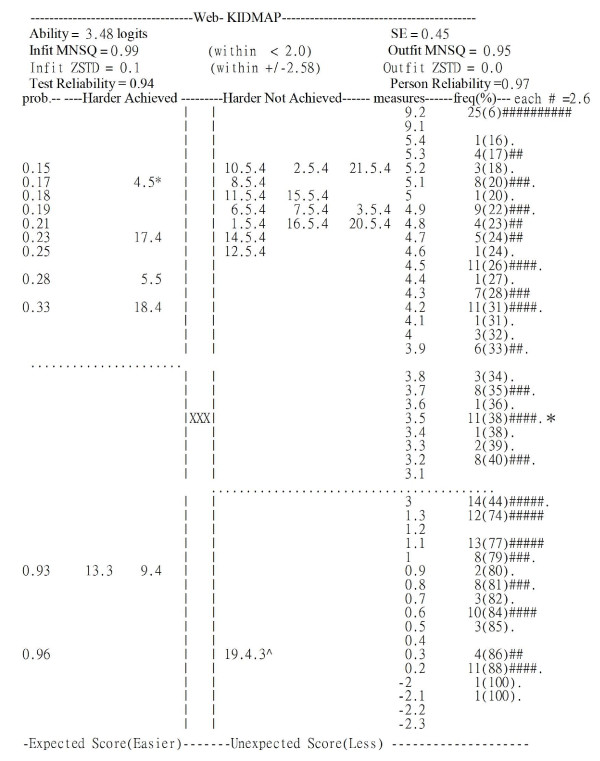
**KIDMAP for patient 2 showing a good fit to the model**. Note: *p < .05; ^ *p *< .01.

Figures 1 and figure 2 present two KIDMAPs, which were generated from the responses of two patients on the website right after the completion of their consultation with a doctor. Patient 1 (Figure 1) had an Infit and Outfit MNSQ of 2.35 and 2.37, respectively, suggesting the response pattern was too aberrant to reveal useful information. For example, there were many items located in the 2nd quadrant (hard items that were unexpectedly achieved) and the 4^th ^quadrant (easy items that were unexpectedly not achieved). Patient 2 (Figure 2) had an Infit and Outfit MNSQ of 0.99 and 0.95, respectively, indicating the response pattern was reliable. Most items were located in the 1^st ^quadrant (hard items were not achieved) and the 3^rd ^quadrant (easy items were achieved).

## Discussions

### Findings

After removing the two miss-fitting items, the remaining 21-item PFQ met the Rasch model's requirements. The test reliability was 0.94. No DIF was found in relation to gender and education; however, there was DIF in relation to both age and disease. The Web-KIDMAP is an effective approach to collecting patient opinions and providing visual and useful feedback information to doctors. Although the use of Internet surveys of patients requires less follow-up in order to achieve the same completion rate as a mailed survey [14], most Internet surveys fail to render instant feedback for doctors. The Web-KIDMAP developed in this study is able to release visual summary immediately after the completion of the consultation with his/her doctor. Furthermore, the fit MNSQ in the Web-KIDMAP allows the user to assess the reliability of the response pattern. A fit MNSQ greater than 2 suggests too much unexplained noise for the survey to be useful [36]. Note that all of the benefits ascribed to the Web-KIDMAP visual representation are subject to the fact that the Rasch model's requirement holds.

Unfortunately, the traditional non-Web KIDMAP is available only from the computer programs Quest [38] and ConstructMap [39], which were developed mainly for researchers and professionals. Given the popularity and familiarity of the Internet for non-academics, there is a great need for a Web-based KIDMAP generator. In this study we invented a computer program that ran on the Internet and yielded a Web-KIDMAP. Details of the estimation methods for item difficulties, person abilities and fit statistics in KIDMAP can be found in Linacre & Wright [40], Wright & Masters [19] and Chien & Wang et al. [41].

### Strengths of the study

**Figure 3:**
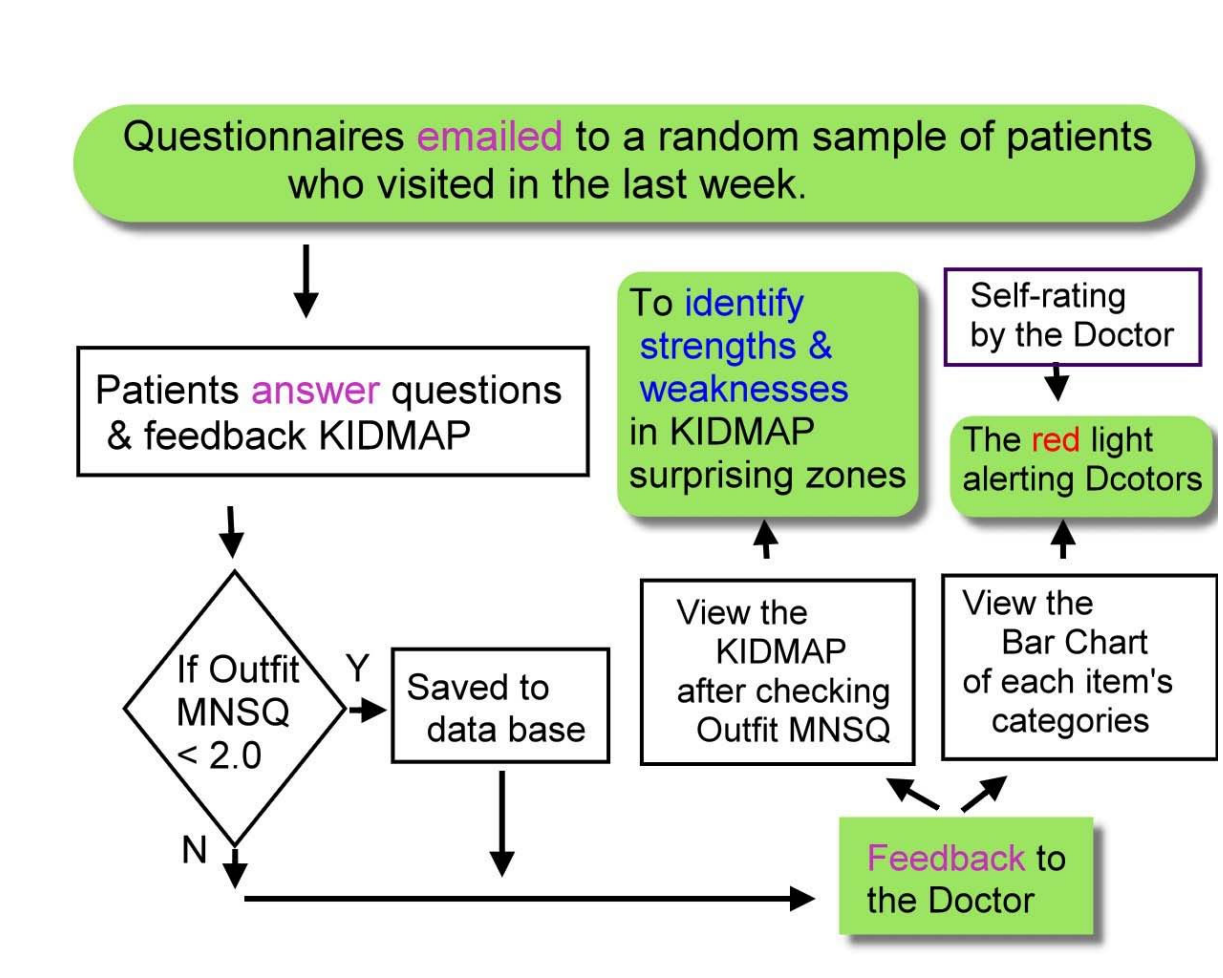
**Steps in the feedback process**.

The Picker Institute Europe recently reviewed a selection of ten questionnaires and identified 23 items that can be classified into five domains [26]. The PFQ has been assessed using methods derived from CTT. However, there are many shortcomings in CTT, including the mutual dependence of item measure and person measure, ordinal raw score rather than interval data, and difficulty in handling missing data. In this study, we applied the Rasch model to analyzing the Patient Feedback Questionnaire. With the use of Rasch analysis, we were able to detect aberrant responses and DIF, and to produce linear measures. In addition, KIDMAP, which is available with Rasch analysis, allows a doctor to self-rate his/her expected performance across items and then compare it with the average scores of each item responded by all patients; this allows them to attain an "always comparing, always improving quality of service" at the physician level through the three steps of feedback process shown in Figure 3.

Another issue worthy of mention is the appropriateness of the scaling level in the PFQ. All category boundary parameters of the PFQ items are ordered, as in the sequence, -3.06, -2.36, 0.44 and 4.97, under the rating scale model, which share the same threshold difficulties. These threshold difficulties are congruent with the guidelines for the rating scales [42] in which calibrations increase monotonically with category number [37,43]. This means that the questionnaire assessing the doctors' performance from the patient viewpoint is appropriate to a 5-point rating scale.

### Limitations of the study

High quality patient feedback is important; clearly further work is still needed to improve the administration of an effective patient feedback tool for a clinical setting. In this study, we explore the questionnaire as a tool to collect an individual's perception using a visual representation like KIDMAP [35]. However, users may need some training in order to interpret a KIDMAP correctly.

There are a number of questionnaires that survey a patient's views of their doctor. Patient-doctor interaction, which may reflect the quality of care delivery at the organizational level, is a key component in these questionnaires. However, in this study we merely focus on the process of patient feedback at the physician level.

### Applications

In this study we transplanted the estimates from the WINSTEPS software into the Web-KIDMAP module http://www.webcitation.org/5VWkRosJn and used this to create a 3-step assessment of doctor performance. First, a doctor completes the self-assessment survey and stores each item response in a database. Second, each patient is invited to fill out the online survey and our system then sends the KIDMAP to their doctor. Finally, the doctor or their staff receives the feedback email that describes Outfit MNSQ and this links with the visual KIDMAP of each patient as well as the grouped results of the whole sample.

Patient 2 (Figure 2) had a very good fit, whereas patient 1 (Figure 1) had a poor fit. The segments for those two patients in the Figures are outlined in Table 3, which shows the various fit statistics, raw scores and measures. In this study, patients with both Infit and Outfit *t *beyond ± 2.58 (*p *< .01) and with a MNSQ greater than 2.0[36] were deemed to be possibly careless, mistaken, awkward when using the system or deceptive when responding to the questionnaire.

**Table 3:** Summary statistics for Patients 1 and 2

No.	Actual Score	Maximum Score	logits	SE	Infit MNSQ	Outfit MNSQ	Infit *t*	Outfit *t*
1	66	105	-0.48	0.36	2.35	2.37	2.94	2.99

2	87	105	3.48	0.45	0.99	0.95	0.10	0.00

The MNSQ value is high for Patient 1, but not for patient 2, which is explained by the large number of unexpected (Z-score beyond ± 1.96) response items in the 2^nd ^and 4^th ^quadrants (11 and 5 items in Figures 1 and 2, respectively). This is obvious from even a quick glance at the unexpected response items above or beneath the dotted line and far from the "XXX" sign in the center column of the KIDMAP. The dotted lines indicate the upper and lower boundaries (ability estimate minus one standard error) of the patient's satisfaction level with a 50% probability of success. Readers may be both surprised and delighted that the MNSQ value can alert those considering a case on quantitative grounds are able to easily differentiate a meaningless (e.g., Figure 1) or a meaningful (e.g., Figure 2) feedback using the KIDMAP, before examining specific items for significance using a Z-score from the patient point of view.

### Further studies and suggestions

Two items that pertained to chronic and preventive services were removed from the PFQ data because they did not meet the Rasch model's expectations and were thus inappropriate for the acute patients used in this study. Further consideration should be given to investigate whether these two items are appropriate for other groups of patents.

Patient questionnaires need to be more attuned to patient-centered healthcare. Rasch analysis should be applied to questionnaires in order to assess their psychometric properties and this should improve the administration and interpretation of patient feedback surveys in a clinical setting.

The revised 21-item PFQ was able to discriminate five strata. Furthermore, the person separation reliability was 0.94. In this context, the latter is positively related to the strata by [= (4 × *seperation_index *+ 1) ÷ 3] [19,37], in which the separation index assesses the degree to which the questionnaire is able to discriminate between individuals. A high discrimination indicates a good quality of measurement [44]. It is clear that researchers are able to present test reliability and person conditional reliability using KIDMAP as seen in Figure 2. In addition, in this context, they will also able to use the Rasch separation index when evaluating and designing new questionnaires

## Conclusion

The developed Web-KIDMAP can be used as an easy, fast and simple way to help patients email feedback on their doctors' performance. Our work supports the hypotheses that the psychometric properties of the revised PFQ online version are valid and reliable, and that the KIDMAP module is worthy of its task.

## List of abbreviations

DIF: differential item functioning; MNSQ: mean square error; IRT: item response; theory; CTT: classic test theory; IVR: interactive voice response

## Competing interests

The authors declare that they have no competing interests.

## Authors' contributions

TW and SB provided concept/ideas/research design, writing, data analysis, facilities/equipment and fund procurement. SB and CY collected data from survey and provided institutional liaisons and project management. WW and HR provided consultation (including English revision and review of the manuscript before submission). All authors read and approved the final manuscript.

## Pre-publication history

The pre-publication history for this paper can be accessed here:

http://www.biomedcentral.com/1471-2288/9/38/prepub

## Supplementary Material

Additional file 1 The 23-item Patient Feedback Questionnaire and its five domains. Click here for file
