# Supplementary material for: KIDMAP, a web based system for gathering patients' feedback on their doctors
Source: BMC Med Res Methodol. 2009 Jun 17;9:38. doi: 10.1186/1471-2288-9-38 (PMC2709634; doi:10.1186/1471-2288-9-38)
Supplement: Additional file 1 — The 23-item Patient Feedback Questionnaire and its five domains. [file 1471-2288-9-38-S1.pdf]

Appendix 1: The 23-item Patient Feedback Questionnaire and its five domains

Interpersonal skills	
1	Instilling confidence and trust, being open.
2	Taking a holistic approach, showing empathy, considering the patient's personal situation and concerns
3	Being 'good with people,' caring and courteous, putting the patient at their ease, not patronizing, taking them seriously.
4	Being positive and reassuring.
5	Demonstrating respect for the patient, their privacy and dignity.
Communication of information	
6	Giving clear, understandable explanations about diagnosis and treatment, and when conducting an examination.
7	Eliciting information from patients about their symptoms and concerns, letting them 'tell their story' and asking about their personal life where appropriate.
8	Being thorough in discussion, encouraging and answering questions.
9	Listening carefully and sympathetically.
Patient engagement and enablement	
10	Helping the patient to understand and cope with their illness or condition, health and treatment.
11	Involving the patient in decisions about care and treatment.
12	Providing advice to enable patients to keep healthy.
13	Providing information to support self-care, for example about how to take medication when to return for follow-up care, test results etc.
14	Helping the patient to access other sources of information or support, for example written information, helpline, websites.
15	Giving information about risk in a clear and comprehensible manner.
16	Promoting health literacy, helping patients build skills to access and interpret information.
Overall satisfaction	
17	Overall levels of satisfaction.
18	Whether the patient would recommend this doctor to a friend or family member.
19	Whether the patient would choose this doctor in future.
Technical competence	
20	Expected/thorough screening or diagnostic procedures were carried out.
21	In physical examinations or procedures levels of pain or discomfort were kept to a minimum.
22	A plan to manage a chronic condition had been developed with the doctor.
23	Expected preventive procedures had been offered (such as a flu vaccination for patients over sixty-five).
